# Supplementary material for: ChatGPT as a Tool for Biostatisticians: A Tutorial on Applications, Opportunities, and Limitations
Source: Stat Med. 2025 Oct 23;44(23-24):e70263. doi: 10.1002/sim.70263 (PMC12548020; doi:10.1002/sim.70263)
Supplement: Supplementary file 1 — Data S1. Supporting Information. [file SIM-44-0-s001.zip › Supplementary Materials Additional.zip!/3.5_Causal_Inferece/3.5_Causal_Chat3.pdf]

Combining statistical and causal thinking_3 - Clear memory request

Please clear memory.

I've cleared the memory of our past interactions. If there's anything you'd like to start fresh with, just let me know!

Take the role of an expert biostatistician for this Chat.

Understood. I'll act as an expert biostatistician for this chat—ready to support you with study design, analysis plans, causal inference, survival analysis, regulatory guidance, or anything else you need. What can I help you with today?

I have data from a randomised clinical trial. I want to compare the efficacy of the experimental treatment with that of the control on overall survival in patients with non-small cell lung cancer. In this two-arm RCT, a considerable number of patients in the control arm switched to the experimental treatment after disease progression, while those in the experimental arm never switched.
My goal is to estimate the causal effect of sustained intake of the experimental treatment versus sustained intake of control on Overall survival, had the control group patients not been allowed to switch. Based on the information provided above, please define my target Estimand, following the guidance from the Estimand framework (ICH-E9 addendum).

Based on your objective and the context provided, here is a structured definition of your **target estimand** using the **ICH E9(R1) estimand framework**, which includes the **five key attributes**: treatment, population, variable (endpoint), intercurrent events, and summary measure.

Target Estimand (Hypothetical Strategy for Treatment Switching)

1. Treatment condition of interest:

- *Experimental intervention:* Sustained treatment with the experimental agent, from randomisation until death or censoring.
- *Control intervention:* Sustained treatment with the control agent, without switching to the experimental treatment after disease progression.

2. Population:

- All randomised patients with non-small cell lung cancer enrolled in the trial (i.e. the **intention-to-treat** population).

3. Variable (endpoint):

- Overall survival (OS), defined as time from randomisation to death from any cause.

4. Intercurrent events and strategies to handle them:

- **Treatment switching from control to experimental arm:**
  Handled using a hypothetical strategy — estimate the effect as if patients in the control group had not switched to the experimental treatment after progression.
- **Other intercurrent events (e.g., treatment discontinuation for other reasons, non-cancer death):**
  Handled using treatment policy strategy, unless otherwise specified, i.e. all such events are included in the outcome definition as they occur during follow-up.

5. Summary measure:

- Difference in restricted mean survival time (RMST) over a pre-specified time horizon
  or
- Hazard ratio (HR) from a suitable causal model (e.g., using inverse probability of censoring weighting, IPCW) comparing the two sustained treatment regimens.

Plain language interpretation:

You aim to estimate the causal effect on overall survival **as if all patients had remained on their originally assigned treatment throughout** (i.e., no switching from control to experimental). This hypothetical estimand enables a clear comparison of the two sustained treatment strategies, despite post-randomisation treatment changes in the observed data.

Would you like help selecting and justifying appropriate statistical methods (e.g. IPCW, RPSFTM, two-stage estimation, cloning/censoring/weighting) to estimate this estimand from your data?

The switching behaviour is affected by a set of different baseline and time-varying covariates. Here, I want to assume that the time-varying confounders that influence the switching behaviour and Overall survival are also themselves affected by prior treatment intake.
For e.g. ECOG score might not only affect whether patients should switch treatment or not, but its value might also be affected by prior treatment intake reflected by the switching behaviour.
In this case, the analytical problem becomes more complex. Can you explain?

Yes, you're describing a **classic time-varying confounding problem with treatment-confounder feedback**, which indeed complicates estimation of causal effects. Let's unpack why.

---

1. Standard methods fail under treatment-confounder feedback

When:

- **Time-varying confounders (e.g. ECOG score)** influence both treatment switching and the outcome (Overall Survival),
- And **these confounders are themselves influenced by prior treatment**,

...you have a **feedback loop**:
Prior treatment → Time-varying confounder (e.g. ECOG) → Future treatment (e.g. switching) → Outcome (OS)

This feedback violates the assumptions of **standard regression or time-dependent Cox models**, because:

- Adjusting for the time-varying confounders in such models would **block part of the treatment effect** mediated through them.
- Failing to adjust for them would result in **confounding bias**, since these confounders affect both treatment and outcome.

Thus, neither approach gives an unbiased estimate of the causal effect of sustained treatment.

---

2. Causal interpretation requires g-methods

To estimate the **causal effect of sustained treatment strategies** under these conditions, you must use **g-methods** designed to handle time-varying confounding affected by prior treatment. The three main options are:

a) Marginal Structural Models (MSMs) using Inverse Probability of Censoring Weights (IPCW)

- Artificially censor patients at the time of switching (to simulate a hypothetical strategy).
- Use IPCW to adjust for **informative censoring**, where the probability of remaining uncensored depends on time-varying confounders.
- Weights must be carefully constructed to reflect the full treatment-confounder feedback.

b) G-computation formula / Sequential Regression

- Explicitly models the joint distribution of time-varying covariates, treatment, and survival time.
- Requires modelling assumptions for the full data-generating process.

c) G-estimation of Structural Nested Models (e.g., Structural Nested Accelerated Failure Time Models)

- Models the causal effect of treatment history on survival, accounting for time-varying confounding and feedback.

Each method requires:

- **Proper specification of time-varying models** (for confounders, treatment switching, and survival).

- Sufficient overlap and no unmeasured confounding.
- Careful handling of positivity violations (i.e., patients must have a nonzero probability of following each treatment regime given their history).

---

3. Summary: Why this complicates the analysis

Challenge	Explanation
**Feedback loop**	Confounders are affected by prior treatment, breaking standard adjustment assumptions.
**Informative switching**	Patients who switch differ systematically in prognosis and health status.
**Bias with standard models**	Naively adjusting for time-varying confounders introduces bias; failing to adjust causes confounding.
**Need for specialised methods**	g-methods are required to appropriately adjust for time-varying confounding with feedback.

Would you like help formulating an appropriate causal DAG for this setting, or specifying an MSM/IPCW approach tailored to your data structure?

I would like to apply the inverse probability of censoring weighting (IPCW) approach to address the bias introduced by switching following the methodology discussed in the following paper: Robins JM, Finkelstein DM. Correcting for noncompliance and dependent censoring in an AIDS clinical trial with inverse probability of censoring weighted (IPCW) log-rank tests. Biometrics. 2000;56(3):779–88.
Briefly summarise the key points from this paper

Certainly. The **Robins & Finkelstein (2000)** paper is foundational in the development of the **Inverse Probability of Censoring Weighting (IPCW)** method to correct for **dependent censoring** and **noncompliance**, such as treatment switching in clinical trials. Here is a summary of its key contributions:

Key Contributions of Robins & Finkelstein (2000)

1. Problem motivation:

- In randomised trials, some patients **deviate from the assigned treatment**, e.g. by **switching treatments** after disease progression.
- These deviations are often **non-random** and depend on **time-varying covariates** that also affect the outcome (e.g. health status).
- Standard survival analysis (e.g. log-rank test or Cox model) may yield **biased estimates** under this dependent treatment switching or censoring.

---

2. Proposed solution: IPCW log-rank tests

- The authors propose a method to **correct for dependent censoring** by weighting each patient's contribution to the test statistic.
- Patients are **artificially censored** at the time they deviate from the protocol (e.g. switch treatments).
- Each uncensored patient is assigned a **weight equal to the inverse of the estimated probability** of remaining uncensored up to that time, conditional on their covariate history.

---

3. Censoring weights:

- These **inverse probability weights** are estimated from a model (e.g. logistic or Cox model) for the censoring process.
- The model includes **baseline and time-varying covariates** that predict switching (i.e. dependent censoring).
- This approach assumes that, **conditional on observed covariates**, censoring is independent of the potential outcome (i.e. **no unmeasured confounding**).

---

4. Application to survival analysis:

- The IPCW-adjusted **log-rank test** and **Cox regression** are proposed to estimate causal treatment effects under hypothetical strategies.
- The method yields **consistent estimates** of the causal effect of the originally assigned treatment, **as if patients had adhered** to their randomised assignment (e.g. no switching).

---

5. Assumptions:

- **Positivity**: There must be a non-zero probability of remaining uncensored (i.e. not switching) at each time point across all covariate strata.
- **Correct model specification**: The model used to estimate censoring probabilities must be correctly specified.
- **No unmeasured confounding** of the switching and survival process.

---

6. Advantages:

- Allows estimation of **causal effects under a hypothetical strategy**, even when protocol deviations like switching are common.
- Handles **time-varying confounding affected by prior treatment**, unlike standard regression or Cox models.

---

7. Practical example:

- The method is illustrated using data from an **AIDS clinical trial**, where patients could switch from placebo to active treatment based on declining CD4 count.

- Without proper adjustment, estimates would be biased due to informative switching.

Conclusion

The paper demonstrates how **IPCW can be used to recover causal estimands** in settings with time-varying, treatment-dependent censoring—such as treatment switching in oncology trials. It lays the groundwork for applying IPCW in modern causal inference with longitudinal data.

Would you like help setting up the censoring model and constructing the weights for your data, or a checklist for applying IPCW in your specific setting?

Could you elaborate more on the research question Robins and Finkelstein were trying to answer in their paper, mentioning the comparator arms, possible intercurrent events, outcome definition and population under study.

Certainly. Here's an elaboration of the **research question** and study context from **Robins & Finkelstein (2000)**, focusing on the key elements aligned with the estimand framework—**population**, **treatments**, **intercurrent events**, and **outcome**—to clarify what problem they were trying to solve.

Research Question

Robins and Finkelstein aimed to estimate the **causal effect of initial randomised treatment assignment** (zidovudine vs. placebo) on **survival** in an AIDS clinical trial, under a hypothetical scenario where all patients had continued with their randomised treatment (i.e. no treatment switching), despite the presence of **informative noncompliance** (switching) that was influenced by **time-varying patient characteristics**.

Study Context and Estimand Components

1. Population

- HIV-positive adults enrolled in a **randomised controlled trial** (the NIAID 505 trial).
- All participants were included in the intention-to-treat population for causal inference.

2. Treatment Conditions (Intervention and Comparator Arms)

- **Experimental arm**: Zidovudine (AZT), an antiretroviral treatment.
- **Control arm**: Placebo.

Patients were initially randomised to one of these two arms.

3. Intercurrent Events

- The **key intercurrent event** was **treatment switching**, specifically:

- Patients originally assigned to placebo were **allowed to switch to zidovudine** if their **CD4 count declined** below a threshold.
- This switching was **not random**, but triggered by **time-varying indicators of health deterioration** (e.g., CD4 count), which themselves may have been influenced by earlier treatment.

- Other potential intercurrent events (e.g., loss to follow-up) were secondary to the main issue of treatment switching.

4. Outcome Variable

- **Overall survival**, defined as **time from randomisation to death** from any cause.
- The goal was to estimate survival under **continued receipt of the initially assigned treatment**, despite observed switching in the data.

5. Estimand / Research Objective

- **Estimand type**: A **hypothetical estimand** targeting the effect that would have been observed **had all patients remained on their originally assigned treatment throughout** follow-up, **regardless of their observed switching** behaviour.
- **Causal contrast**: The **difference in survival distributions** (or hazard ratios) between sustained zidovudine and sustained placebo use, correcting for **non-random treatment switching**.
- The authors used IPCW to simulate a world where **treatment switching did not occur**, by censoring at switch time and applying **inverse probability weights** to correct for dependent censoring.

Challenge Addressed

- **Naive survival analysis** (e.g., ITT or per-protocol) would be biased:
  - ITT ignores switching and underestimates the effect of zidovudine.
  - Per-protocol or naive as-treated analyses would adjust for time-varying covariates affected by prior treatment, which leads to **bias due to collider stratification or mediator blocking**.

Thus, the IPCW approach enabled **causal estimation in the presence of time-varying confounding and noncompliance**—a major methodological advancement for longitudinal trials with dynamic treatment strategies.

Let me know if you'd like a schematic DAG for this setup or a translation of this problem structure to your oncology trial context.

Can you depict our research problem in a DAG?

Absolutely. Here's a **Directed Acyclic Graph (DAG)** that captures the causal structure of your research problem, which involves:

- **Treatment switching** in the control arm after progression
- **Time-varying confounders** (e.g. ECOG score, disease progression) that both influence and are influenced by treatment
- **Overall survival** as the outcome

Variables

- **A(0)**: Initial treatment assignment (randomised: experimental vs. control)
- **L(0)**: Baseline covariates (e.g. age, stage, smoking history)
- **L(1), L(2), ..., L(t)**: Time-varying covariates (e.g. ECOG score, progression, lesion size)
- **A(1), A(2), ..., A(t)**: Treatment at time t (e.g. switching behaviour—remains on control or switches to experimental)
- **Y**: Overall survival (event time)

Text-based DAG (simplified)

```plaintext
        L(0)
        /  \
      /      \
   A(0)     L(1) <--------
     \       |           |
      \      v           |
       ---> A(1) ---> Y  |
                ^        |
                |        |
              L(2) <----
```

Explanation of Arrows

- **A(0) ← L(0)**: Randomisation is independent of covariates (but covariates can affect treatment effect heterogeneity).
- **L(t) ← A(t-1)**: Time-varying covariates are affected by prior treatment (e.g. ECOG worsens less under active treatment).
- **A(t) ← L(t)**: Treatment decisions (e.g. switching) are based on evolving health status.
- **Y ← A(t), L(t)**: Both treatment history and covariates affect survival.

Key Feature: Treatment-Confounder Feedback

- **L(t) is both a confounder and mediator**:
  - It affects future treatment decisions (switching),
  - But is also affected by past treatment.

This creates a **feedback loop**, which **biases standard regression or time-dependent Cox models** if not handled correctly.

Estimation Implication

To estimate the **causal effect of sustained treatment strategies** (e.g. always experimental vs. always control), we must:

- Avoid **conditioning on L(t)** in conventional models.
- Use **IPCW or other g-methods** to adjust for the **informative censoring** introduced by switching, while respecting the time-varying nature of confounding.

Would you like a graphical DAG image (PDF or PNG), or code to generate this in `dagitty` or `ggdag`?

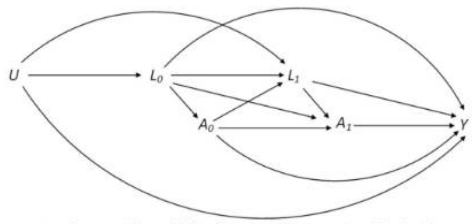

Fig. 1 Graphical representations of our research focus and the bias of naive methods for time-varying confounding in the presence of treatment-confounder feedback: This Directed acyclic graph (DAG) represents the post-progression period for patients in the control arm. Following disease progression, the decision as to whether or not a patient in the control group continues to take its treatment (A_0) or (A_1) depends on predictive factors measured at or before the time of disease progression (L_0). Again, we assume that treatment intake (A_0) affects outcome (Y) and influences a set of TVCs at a later time-point (L_1). These covariates are also assumed to impact the clinician's decision as to whether or not a patient should subsequently change treatments (A_1) introducing time-varying confounding and treatment-confounder feedback through L_1

Attached, I provide you with a directed acyclic graph (DAG) that represents our research problem.
To simplify this DAG and take into consideration this deterministic information provided below, I restrict my DAG to the post-progression period for patients in the control arm only and consider two follow-up time points, 1 & 2.
Here A(k) is deterministically 0 if A(k-1)=0 AND L(k-1) (reflecting progression status)=0 (no progression).
Following disease progression, the decision as to whether or not a patient in the control group continues to take their treatment (A0 or A1) depends on predictive factors measured at or before the time of disease progression (L0).
Again, we assume that treatment intake (A0) affects outcome (Y) and influences a set of TVCS at a later time-point (L1).
These covariates are also assumed to impact the clinician's decision as to whether or not a patient should subsequently change treatments (A1), introducing time-varying confounding and treatment confounder feedback through L1.
I will emphasise again that in our case scenario, patients in the control group do not switch unless they progress, and all patients in the experimental group never switch.
I still want to estimate in my outcome model the causal effects of sustained treatment interventions (always taking control versus always taking experimental) under a hypothetical strategy where control patients do not switch treatment.
I want you to use this DAG as a basis for performing the appropriate IPCW analyses and provide me with the analysis R-script.
You may assume that the dataset is in long format, with variables: id, time (in months and increases

with increments of 1), death indicator, switching indicator, baseline covariates repeated over time points, time-varying covariates at each time pt.

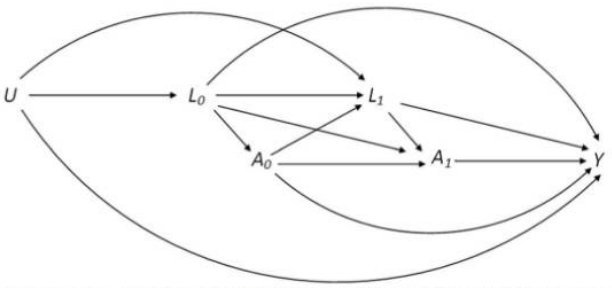

Fig. 1 Graphical representations of our research focus and the bias of naive methods for time-varying confounding in the presence of treatment-confounder feedback: This Directed acyclic graph (DAG) represents the post-progression period for patients in the control arm. Following disease progression, the decision as to whether or not a patient in the control group continues to take its treatment (A_0) or (A_1) depends on predictive factors measured at or before the time of disease progression (L_0). Again, we assume that treatment intake (A_0) affects outcome (Y) and influences a set of TVCs at a later time-point (L_1). These covariates are also assumed to impact the clinician's decision as to whether or not a patient should subsequently change treatments (A_1) introducing time-varying confounding and treatment-confounder feedback through L_1

Thank you for the detailed description and DAG. Based on your setup:

- You want to estimate the **causal effect of sustained treatment** (experimental vs. control) on survival,
- Patients in the **control arm may switch only after progression**, and
- There is **time-varying confounding** with feedback (e.g. prior treatment influences L1, which affects switching).

This justifies an **IPCW approach**, where we:

1. **Artificially censor** control patients at the time they switch,
2. Use **inverse probability of censoring weights** to adjust for the informative nature of this artificial censoring.

🔧 R Code: IPCW Implementation for Your Setting

This script assumes your data is in **long format** with variables:

- `` `id` ``: patient identifier
- `` `time` ``: months since randomisation
- `` `death` ``: event indicator (1 = death)
- `` `switch` ``: switching indicator (1 = switched at this time)
- `` `A` ``: treatment at time `` `t` `` (0 = control, 1 = experimental)
- `` `L0_*` ``: baseline covariates (e.g., age, ECOG, etc.)
- `` `L_tvc` ``: time-varying covariates (e.g., ECOG, lesion size) at time `` `t` ``

```r
# Required packages
library(survival)
library(dplyr)
library(ipw)
```

```
# Step 1: Artificial censoring at time of switch in the control group
data_ipcw <- your_data %>%
  group_by(id) %>%
  mutate(
    switch_time = min(time[switch == 1], na.rm = TRUE),
    censored = ifelse(time >= switch_time & A == 0, 1, 0),
    event = ifelse(censored == 1, 0, death)  # censor at switch time
  ) %>%
  ungroup()

# Step 2: Fit the censoring model (logistic model for probability of not being censored)
# Weights are estimated for control patients only (A == 0)
data_ipcw <- data_ipcw %>%
  mutate(not_censored = 1 - censored)

censor_model <- glm(
  not_censored ~ L0_age + L0_ECOG + L_tvc + time,  # add all relevant baseline & time-varying covar
  family = binomial(),
  data = filter(data_ipcw, A == 0)
)

# Predict probabilities and compute weights
data_ipcw <- data_ipcw %>%
  mutate(
    pred_survival_prob = predict(censor_model, type = "response"),
    weight = ifelse(A == 0, 1 / pred_survival_prob, 1)  # weights only needed for control group
  )

# Step 3: Fit weighted survival model (IPCW-adjusted)
surv_fit <- coxph(
  Surv(time, event) ~ A,  # compare sustained A = 1 vs A = 0
  data = data_ipcw,
  weights = weight,
  robust = TRUE
)

summary(surv_fit)
```

🧠 Key Assumptions

- The censoring model is correctly specified and includes all confounders of switching and survival.
- No unmeasured confounding.
- Positivity: Patients must have a non-zero probability of remaining uncensored in all strata.

🔄 Next Steps

- Review covariates in your switching model (`` `censor_model` ``)—include all L0 and time-varying L(t) that predict switching and survival.
- Assess weight stability (e.g. plot distribution of weights, check for extreme values).
- Consider truncating extreme weights if necessary.

Would you like me to help you adapt this script to a real dataset or simulate a small dataset to test it?
